# Diagnostic yield and financial implications of a nationwide electrocardiographic screening programme to detect cardiac disease in the young

**DOI:** 10.1093/europace/euab021

**Published:** 2021-02-11

**Authors:** Harshil Dhutia, Aneil Malhotra, Gherardo Finocchiaro, Sameer Parpia, Raghav Bhatia, Andrew D’Silva, Sabiha Gati, Greg Mellor, Rajay Narain, Navin Chandra, Elijah Behr, Maite Tome, Michael Papadakis, Sanjay Sharma

**Affiliations:** Department of Cardiology, Glenfield Hospital, University Hospitals of Leicester, Leicester, UK; Cardiology Clinical Academic Group, Molecular and Clinical Sciences Research Institute, St. George's, University of London University of London, UK; Cardiology Clinical Academic Group, Molecular and Clinical Sciences Research Institute, St. George's, University of London University of London, UK; Division of Cardiovascular Sciences, University of Manchester and Manchester Foundation NHS Trust, Manchester, UK; Cardiology Clinical Academic Group, Molecular and Clinical Sciences Research Institute, St. George's, University of London University of London, UK; Ontario Clinical Oncology Group, McMaster University, Hamilton, ON, Canada; Cardiology Clinical Academic Group, Molecular and Clinical Sciences Research Institute, St. George's, University of London University of London, UK; Cardiology Clinical Academic Group, Molecular and Clinical Sciences Research Institute, St. George's, University of London University of London, UK; Cardiology Clinical Academic Group, Molecular and Clinical Sciences Research Institute, St. George's, University of London University of London, UK; Cardiology Clinical Academic Group, Molecular and Clinical Sciences Research Institute, St. George's, University of London University of London, UK; Cardiology Clinical Academic Group, Molecular and Clinical Sciences Research Institute, St. George's, University of London University of London, UK; Cardiology Clinical Academic Group, Molecular and Clinical Sciences Research Institute, St. George's, University of London University of London, UK; Cardiology Clinical Academic Group, Molecular and Clinical Sciences Research Institute, St. George's, University of London University of London, UK; Cardiology Clinical Academic Group, Molecular and Clinical Sciences Research Institute, St. George's, University of London University of London, UK; Cardiology Clinical Academic Group, Molecular and Clinical Sciences Research Institute, St. George's, University of London University of London, UK; Cardiology Clinical Academic Group, Molecular and Clinical Sciences Research Institute, St. George's, University of London University of London, UK

**Keywords:** Sudden cardiac death, Electrocardiography, Screening, Young

## Abstract

**Aims:**

There is limited information on the role of screening with electrocardiography (ECG) for identifying cardiovascular diseases associated with sudden cardiac death (SCD) in a non-select group of adolescents and young adults in the general population.

**Methods and results:**

Between 2012 and 2014, 26 900 young individuals (aged 14–35 years) were prospectively evaluated with a health questionnaire and ECG. Individuals with abnormal results underwent secondary investigations, the costs of which were being based on the UK National Health Service tariffs. Six hundred and seventy-five (2.5%) individuals required further investigation for an abnormal health questionnaire, 2175 (8.1%) for an abnormal ECG, and 114 (0.5%) for both. Diseases associated with young SCD were identified in 88 (0.3%) individuals of which 15 (17%) were detected with the health questionnaire, 72 (81%) with ECG and 2 (2%) with both. Forty-nine (56%) of these individuals received medical intervention beyond lifestyle modification advice in the follow-up period of 24 months. The overall cost of the evaluation process was €97 per person screened, €17 834 per cardiovascular disease detected, and €29 588 per cardiovascular disease associated with SCD detected. Inclusion of ECG was associated with a 36% cost reduction per diagnosis of diseases associated with SCD compared with the health questionnaire alone.

**Conclusion:**

The inclusion of an ECG to a health questionnaire is associated with a five-fold increase in the ability to detect disease associated with SCD in young individuals and is more cost effective for detecting serious disease compared with screening with a health questionnaire alone.

## Graphical Abstract

**Figure:**
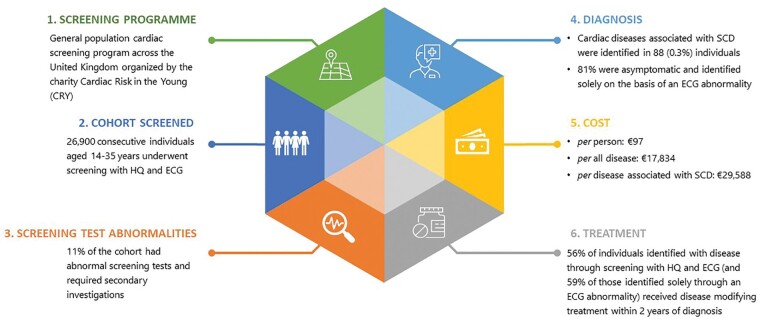

**What’s new?**

- This is the first study to report the results of an electrocardiogram-based cardiovascular screening programme in the young general population at a nationwide level.
- The prevalence of cardiovascular disease associated with young sudden cardiac death in the general population is 0.3%.
- The cost of electrocardiogram-based cardiovascular screening is €97 per person screened, €17 834 per cardiovascular disease detected and €29 588 per cardiovascular disease associated with sudden cardiac death detected.
- Inclusion of an electrocardiogram to the current practice of symptom and family history driven evaluation increases the diagnostic yield for serious cardiac disease by five-fold and is associated with a 36% reduction in cost per disease detected.
- Most young individuals identified with serious cardiac disease (including those who are asymptomatic and have no worrying family history) received disease modifying therapy within 2 years of detection through screening.

## Introduction

Most sudden cardiac deaths (SCD) in young individuals are due to hereditary or congenital heart diseases that are detectable during life and the natural history and risk posed by these diseases can be modified through several established medical interventions. Evidence for the efficacy of cardiovascular screening to identify young individuals with cardiac disease associated with SCD is derived exclusively from young competitive athletes.^1^^,^^2^ Although the incidence of SCD in young athletes is higher than in non-athletes, the ethics of limiting cardiac screening to competitive athletes are questionable because non-athletes represent a significantly larger group in whom the absolute number of deaths is higher than in athletes.^3^^,^^4^ The National Health Service (NHS) in the UK acknowledges the importance of identifying all young individuals at risk of SCD, however, cardiovascular evaluation is currently limited to those reporting symptoms or a family history of cardiovascular disease and those with abnormal findings during incidental cardiovascular examination.^5^ In this first study of its kind, we sought to determine the diagnostic yield and actual financial cost incurred to detect cardiovascular disease through electrocardiography (ECG) screening in a nationwide screening programme in young individuals in the UK.

## Methods

### Setting

The charitable organisation Cardiac Risk in the Young (CRY) facilitates screening for diseases predisposing to SCD in young individuals (aged 14–35 years-old). Such evaluations are accessible to all individuals, irrespective of athletic status, symptoms, or family history of premature cardiac disease. Screening events are advertised in the local media and on the CRY website (www.c-r-y.org). Individuals from the general population self-present to screening events. The evaluations and their reporting are the overall responsibility of the senior author.

### Subjects

Between 2012 and 2014, 27 458 consecutive individuals aged 14–35 years-old self-presented for cardiovascular evaluation comprising a health questionnaire and 12-lead ECG which was conducted by cardiologists experienced in inherited cardiac diseases. Five hundred and fifty-eight (2%) individuals were excluded due to a pre-existing cardiac diagnosis or prior cardiovascular assessment within the past 2 years leaving 26 900 individuals for inclusion in the study. Ethnicity/race was self-reported.

### Screening protocol

#### Health questionnaire

The health questionnaire enquired about cardiac symptoms, past medical history, and family history of premature (<50 years old) cardiac disease or SCD (Supplementary material online, *Figure S1*).

#### Electrocardiography

A resting 12-lead ECG was performed using a Philips Pagewriter Trim III recorder (Philips, Bothell, WA, USA) with a paper speed of 25 mm/s and amplification of 0.1 mV/mm. The absolute QT was corrected for heart rate using the Bazett’s formula.^6^ The Fredericia formula was used at extremes of heart rate. The European Society of Cardiology (ESC) recommendations were used to interpret the ECG as these were derived from 30 000 young individuals who were screened before entering competitive sport and resembled our cohort most closely.^7^ We used slightly longer QT interval cut-offs (>460 ms) as our previous experience has shown that 6.5% of the young non-athlete cohort has a QT above 440 ms in males and 460 ms in females.^8^ We also used a more stringent cut-off of <330 ms to define a short QT interval.^9^

### Further investigations and disease identification

Further investigations were determined by the screening cardiologists. Individuals requiring secondary investigations were referred to local hospitals through their primary care physician with a report that specified the abnormal findings, copy of the ECG, diagnosis in question, and a proposed investigation protocol based on our experience of investigating athletes and young individuals with cardiovascular disease.

Secondary investigations were conducted by local cardiologists who also determined the type and number of secondary investigations. Data relating to secondary investigations and the final diagnosis were obtained from the primary care physician 24 months following the initial evaluation. Diseases considered to be associated with SCD were as previously reported.^10^^,^^11^

### Financial analysis

Costs were incurred in British pounds (£) but presented as Euros (€) at a conversion rate of £1=€1.13 at the time of the manuscript was prepared. The initial investigations (health questionnaire and ECG) were performed at a subsidized cost of €57 per individual. The cost of secondary investigations was based on the UK National Health Service tariff payment system (www.england.nhs.uk/pay-syst/national-tariff) (Supplementary material online, *Table S1*). There is no national rebate for pharmacological testing for Brugada syndrome, tilt table testing, 24-h blood pressure monitoring, or signal average ECG, therefore, we used the fee for these procedures at our institute for the analysis. The fees for genetic testing were derived from the NHS UK genetic testing network.

#### Estimation of cost with a health questionnaire only strategy

When estimating the costs of the health questionnaire only strategy, we assumed an initial screening cost of €17, after accounting for the cost of ECG in the NHS (€40).

### Statistical analysis

Statistical analysis was performed using SPSS. Results are reported as mean ± SD for continuous variables or numbers of cases and percentages for categorical variables with 95% confidence intervals as appropriate. Comparison of groups was performed using the Student’s *t*-test for continuous variables with correction for unequal variance when necessary and χ^2^ test or Fisher’s exact test, as appropriate for categorical variables. Cohen’s kappa (*κ*) coefficient was used to calculate the inter-observer agreement in ECG interpretation.

### Ethics

Ethical approval was granted by the Essex 2 Research Ethical Committee. Written consent was obtained from individuals ≥16 years of age and from a parent/guardian for those <16 years of age.

## Results

### Study participants

Individuals were aged 19.4 ± 4 years. The majority were male (*n* = 17 530; 65%) and white (*n* = 24 299; 90%). Five hundred and forty-six (2%) were of African or Afro-Caribbean origin and 2055 (8%) consisted of other ethnicities. The cohort exercised for 3.9 ± 3.0 h/week. Most (81%) evaluations were undertaken in England including 43 of the 48 lieutenancy counties, 13% in Northern Ireland, 3% in Scotland, and 3% in Wales. Evaluations took place in 214 different venues and occurred predominantly at community centres (71.9%), but also in high schools (19.5%) and healthcare provider centres (hospitals and family practice centres) (8.6%).

### Health questionnaire and electrocardiographic abnormalities

Five thousand four hundred and seventy three [20.4% (95% CI 19.9–20.8%)] individuals reported cardiac symptoms [*n* = 4618 (17.2%)], a family history of cardiovascular disease or premature death [*n* = 641 (2.4%)], or both [*n* = 214 (0.8%)] (*Table 1*). Following consultation with the screening cardiologist, the number considered to have symptoms compatible with cardiac disease and those considered to have a family history suggestive of an inherited cardiac disease was reduced to 381 (1.4%) and 348 (1.3%), respectively. A random sample of 2500 ECGs was reported by two cardiologists blind to other clinical details, with good inter-observer agreement [*κ* = 0.62 (95% CI 0.54–0.70)] for classifying an ECG as abnormal. Sixty (0.2%) individuals had abnormal symptoms and family history. An abnormal ECG was noted in 2289 [8.5% (95% CI 8.1–8.9%)] individuals (*Table 2*).

**Table 1:** Proportion of individuals with cardiovascular symptoms and family history on health questionnaire

	Total positive before physician evaluation (%)	Total positive after physician evaluation (%)
Personal history		
Chest pain	753 (2.7)	31 (0.1)
Palpitations	1141 (4.2)	79 (0.3)
Syncope/pre-syncope	1790 (6.7)	62 (0.2)
Excessive exertional and unexplained fatigue/dyspnoea	506 (1.8)	58 (0.2)
≥1 of above symptoms	642 (2.4)	211 (0.8)
Total	4832 (17.9)	441 (1.6)
Family history		
Premature death—sudden and unexpected before age 50 years due to heart disease, in one or more relatives	426 (1.6)	189 (0.7)
Specific knowledge of certain cardiac conditions in family members	429 (1.6)	219 (0.8)
Total	855 (3.2)	408 (1.5)

**Table 2:** ECG abnormalities

ECG abnormality	Frequency (%)	ECG abnormality	Frequency (%)
≥1 ECG abnormality	2289 (8.5)	Non-specific intraventricular conduction delay	117 (0.4)
T-wave inversion	1062 (3.9)	Right bundle branch block	34 (0.1)
ST depression	30 (0.1)	Left bundle branch block	3 (0.01)
Q-waves	49 (0.2)	Long QT interval	444 (1.6 )
Right axis deviation	298 (1.1)	Short QT interval	5 (0.02)
Left axis deviation	371(1.4)	Pre-excitation	42 (0.2)
Right atrial enlargement	71 (0.3)	Ventricular ectopy	113 (0.4)
Left atrial enlargement	111 (0.4)	Atrial arrhythmia	6 (0.02)
Right ventricular hypertrophy	107 (0.4)	Significant bradyarrhythmia	10 (0.04)

ECG, electrocardiography.

### Follow-up and further investigations

Following preliminary evaluation, 2964 [11.0% (95% CI 10.5–11.6%)] individuals required further investigation including 675 (2.5%) for an abnormal health questionnaire, 2175 (8.1%) for an abnormal ECG, and 114 (0.4%) for both.

Follow-up information pertaining to secondary investigations and diagnoses was available in 2917 (98.5%) individuals. Twenty-five cases with an abnormal health questionnaire and 22 with an abnormal ECG were lost to follow-up.

Transthoracic echocardiography was performed in 2860 (10.6%) individuals, 483 (1.8%) underwent exercise stress testing, 486 (1.8%) underwent Holter monitoring, and 233 (0.9%) underwent cardiac magnetic resonance imaging (Supplementary material online, *Table S2*). Additionally, 115 (0.4%) individuals underwent a combination of 24-h blood pressure monitoring, signal average ECG, tilt table testing electrophysiological studies, trans-oesophageal echocardiography, computed tomography, myocardial perfusion scanning, or pharmacological provocation testing for Brugada syndrome to confirm (or refute) diagnosis of cardiac disease.

### Detection of cardiovascular disease

#### Cardiovascular disease associated with sudden cardiac death

**Figure 1:**
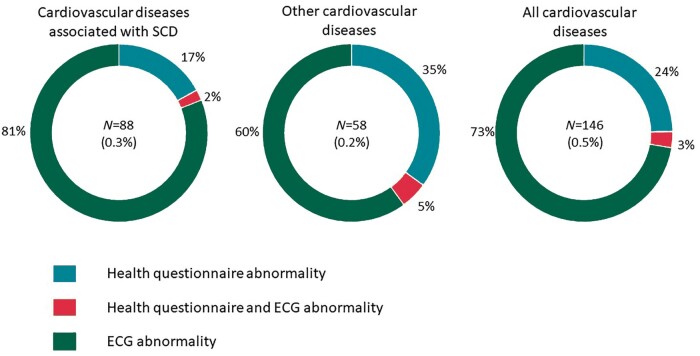
Method of disease detection through cardiovascular screening. Overall, 73% of all diseases and 81% of diseases associated with sudden cardiac death were identified on the basis of an ECG abnormality in asymptomatic individuals with a normal family history. SCD, sudden cardiac death; ECG, electrocardiography.

Cardiac disease potentially associated with SCD was detected in 88 [0.3% (95% CI 0.2–0.4%)] individuals (*Figure 1* and Supplementary material online, *Table S3*). Ventricular pre-excitation [*n* = 42 (48%)], hypertrophic cardiomyopathy [*n* = 14 (16%)], long QT syndrome [*n* = 10 (11%)], and Brugada syndrome [*n* = 8 (9%)] were the most common diagnoses. The other 14 (16%) diseases included Marfan syndrome (*n* = 3), arrhythmogenic right ventricular cardiomyopathy (*n* = 3), dilated cardiomyopathy (*n* = 3), left ventricular non compaction cardiomyopathy (*n* = 2), catecholaminergic polymorphic ventricular tachycardia (*n* = 2), and congenital complete heart block (*n* = 1) (Supplementary material online, *Table S3*). Fifteen (17%) cases were identified by the health questionnaire, 2 (2%) by health questionnaire and ECG, and 71 (81%) solely by ECG (*Figure 1*).

The diagnosis of a long QT syndrome (n=9) following assessment for a prolonged QT was based of a Schwartz score ≥3.5.^12^ All eight cases of Brugada syndrome had a normal baseline ECG and required with an ajmaline provocation test to investigate a family history of Brugada syndrome in a first-degree relative, or SCD. None of the five individuals with a short QT interval were diagnosed with short QT syndrome based on the absence of symptoms, family history, or cardiac arrhythmias during an exercise test or 24-h Holter monitoring period.^13^

#### Other cardiovascular disease

Congenital valvular and septal defects were detected in 58 [0.2% (95% CI 0.2–0.3%)] individuals (Supplementary material online, *Table S4*). Of these, 20 (35%) individuals reported symptoms , 3 (5%) reported symptoms and had an abnormal ECG, and 35 (60%) were asymptomatic but had an abnormal ECG.

In total, a cardiovascular disorder was detected in 146 [0.5% (95% CI 0.5–0.6%)] individuals of which 35 (24%) were identified by the health questionnaire, 5 (3%) by a combination of the health questionnaire and ECG, and 106 (73%) by ECG alone (*Figure 1*).

### Management of individuals identified with potentially serious cardiac disease

During the 24-month follow-up period after screening, 49/88 (56%) individuals diagnosed with potentially serious cardiac disease through the screening programme received ≥1 medical intervention beyond lifestyle advice (Supplementary material online, *Table S3*). Specifically, 22/88 (25%) were prescribed pharmacotherapy, 3/88 (3%) received an implanted cardiac device [pacemaker/implantable cardioverter-defibrillator (ICD)] and 26/88 (30%) underwent ablation.

Medical intervention was implemented in 42/71 (59%) asymptomatic individuals with potentially serious cardiac disease diagnosed solely on the basis of an abnormal ECG. This included two patients who received an ICD for primary prevention during the follow-up period (one patient with arrhythmogenic right ventricular cardiomyopathy and one patient with left ventricular non-compaction cardiomyopathy).

### Financial analysis

#### Total cost of the screening programme

The overall cost of the programme was €2 603 742, equating to a cost of €97 per person screened, €17 834 per cardiovascular disease detected, and €29 588 per cardiac disease associated with SCD detected (*Table 3*).

**Table 3:** Summary of cost incurred to identify cardiovascular disease

Cost	Health Questionnaire and ECG (€)	Health Questionnaire only (€)^a^
Screening cost (*n* = 26 900)	1, 533, 300	457, 300
Cost of additional Investigations following screening		
HQ abnormalities (*n* = 675)	266, 740 (95% CI 227, 012–328, 427)	266, 740 (95% CI 227,012–328, 427)
HQ and ECG abnormalities (*n* = 114)	60, 699 (95% CI 40, 251–91, 313)	60, 699 (95% CI 40, 251–91, 313)
ECG abnormalities (*n* = 2175)	743, 003 (95% CI 700, 492–791, 632)	N/A
Total cost	2 603 742 (95% CI 2 501 055–2 744 672)	784, 739 (95% CI 724, 563–877, 040)
Cost per person screened	97 (95% CI 93–102)	29 (95% CI 27–33)
Cost per cardiovascular disease detected	17, 834 (95% CI 17, 131–18, 799)	19, 618 (95% CI 18, 114–21, 926)
Cost per cardiovascular disease associated with SCD detected	29, 588 (95% CI 28, 421–31, 189)	46, 161 (95% CI 42, 621–51, 591)

a Estimated cost.

ECG, electrocardiography; HQ, health questionnaire; SCD, sudden cardiac death.

#### Estimated cost of health questionnaire only screening strategy

Based on the preliminary evaluation cost of €17 for a health questionnaire, and costs of subsequent investigations following consultation with a cardiologist, the overall cost of screening with a health questionnaire only strategy would have amounted to €784 739 at a cost of €29 per person screened (*Table 3*). This strategy would have identified 40/146 (27%) individuals with all cardiovascular diseases at a cost of €19 618 per disease, and 17/88 (19%) individuals with cardiac disease associated with SCD at a cost of €46 161 per disease.

## Discussion

### Principle findings

We evaluated the diagnostic yield and financial implications of a cardiovascular screening programme comprising of a health questionnaire and 12-lead ECG in almost 27 000 predominantly asymptomatic young individuals in the UK. Subsequent investigations led to the detection of cardiovascular abnormalities in 146 (0.5%) individuals, including 88 (0.3%) with diseases associated with SCD.

As far as we are aware, this is the first study to report the prevalence of cardiovascular disease in the young general population at nationwide level. These results are comparable to the prevalence of diseases reported by cardiovascular screening programmes in competitive athletes.^14–16^ This finding is not surprising as most of the diseases capable of causing SCD in young individuals have a genetic or congenital basis and do not have a unique predilection for competitive sport.

This is also the first study to report the actual financial cost incurred to detect cardiovascular disease through ECG screening in a nationwide program, which amounts to €97 per person screened, €17 834 per cardiovascular disease detected, and €29 588 per cardiac disease associated with SCD detected.

### Disease detection and impact on management

The majority [61/88 (69%)] of diseases identified by screening [including 49/71 (69%) those detected solely on the basis of an abnormal ECG ] were hereditary ion channel diseases or congenital accessory pathways. These conditions are characteristically associated with a structurally normal heart at post-mortem and hence may have important clinical significance since unexplained SCD with a normal macroscopic and histological appearance of the heart at post-mortem is the leading cause of SCD in young individuals.^10^^,^^11^^,^^17^

The identification of individuals with diseases associated with SCD through screening has the potential for several other interventions and risk stratification to minimize the risk of sudden cardiac arrest. In this study, early medical intervention beyond lifestyle modification was implemented in over half of the individuals diagnosed with disease associated with SCD, including ∼60% of those identified solely on the basis of an abnormal ECG (Supplementary material online, *Table S3*). Although only two individuals in the study received an ICD in the short follow-up period, the early identification of disease offers the opportunity for closer clinical surveillance and escalation of treatment in the event of developing high-risk factors in the future.

### Study implications

Cardiovascular evaluation for young individuals in the UK and most western countries is limited to the minority with symptoms or a family history suggestive of cardiovascular disease. In this study, 73% of all individuals with cardiac disease and 81% diagnosed with diseases associated with SCD were asymptomatic or, did not have a relevant family history and would not have been identified through the current healthcare policy (*Figure 1*). Although the overall cost of screening with the ECG is higher, this strategy was associated with a 9% lower cost per all cardiovascular disease detected and a 36% lower cost per cardiac disease associated with SCD detected when compared with screening with the health questionnaire only strategy (*Table 3*).

These findings are likely to have important implications for health policy makers when considering optimal strategies to identify young and apparently healthy individuals with potentially serious cardiac diseases. The results are also significant for physicians as they highlight the diagnostic limitations and potential cost inefficiencies of reliance on symptoms or a family history when deciding to investigate young individuals for cardiac disease.

### Healthcare analytic perspective

It is important to highlight that the financial aspect of this study is limited solely to the cost of disease detection and not on cost-effectiveness of ECG screening in preventing SCD. The aim of our study was to provide a reference for the diagnostic yield and subsequent costs of confirming a serious cardiac diagnosis. It could be argued that the priority of any healthcare system is to focus largely on the highest risk groups. In the Western world, most cardiovascular deaths occur in individuals above 50 years old and are due to atherosclerotic coronary artery disease and heart failure. Therefore, a screening cost of €97 per person for all adolescents and young adults (total number of 16.7 million persons in the UK age 14–35 equating to a cost of €1.62 billion) may be considered as excessive when only 0.5% of all young SCDs occur in this population. Conversely, early identification of a young person with serious cardiac disease has the potential for saving several decades of life through relatively minimal intervention.

### Limitations

This study has several limitations. Investigation beyond preliminary screening was limited to individuals with abnormal symptoms, family history, or ECG; therefore, we could not calculate the sensitivity or specificity of the programme for detecting cardiac disease. We did not perform cardiovascular examination on our patients, therefore we may have failed to detect a number of patients with congenital valvular abnormalities; however current literature suggests that physical examination is associated with a low diagnostic yield especially in young individuals harbouring diseases associated with SCD.^14^^,^^18^ As with any voluntary screening programme, there is the potential for inherent selection bias; however, this is partly mitigated by the large population size and the nationwide enrolment. The cost analysis was based on a subsidized preliminary assessment and relatively modest costs of secondary investigations in the UK National Health Service which are cheaper than other European and North American healthcare models. Screenings were conducted by cardiologists who were highly experienced in inherited cardiac diseases hence there is a possibility that the proportion of individuals referred for further evaluation was lower than if they had been conducted by less experienced healthcare providers. Secondary investigations were at the discretion of the attending cardiologist and may have been influenced by personal clinical practice as with any real-life clinical situation. Finally, the diagnostic yield of serious disease was probably underestimated due to the inherent limitations of the resting ECG for identifying anomalous coronary origins, premature atherosclerotic coronary artery disease, adrenergically mediated arrhythmias, concealed accessory pathways, and incomplete expressions of cardiomyopathy.^19^

## Conclusions

The prevalence of diseases associated with young SCD identified through a cardiovascular screening programme in the general population is 0.3% at a cost of €29 588 per disease detected. The addition of the ECG to health questionnaire improves the ability to detect disease associated with SCD by five-fold, with the majority receiving disease modification therapy within 2 years of diagnosis.

## Supplementary material

Supplementary material is available at *Europace* online.

## Supplementary Material

euab021_Supplementary_Data
