# Supplementary material for: Diagnostic yield and financial implications of a nationwide electrocardiographic screening programme to detect cardiac disease in the young
Source: Europace. 2021 Feb 11;23(8):1295–301. doi: 10.1093/europace/euab021 (PMC8350863; doi:10.1093/europace/euab021)
Supplement: euab021_Supplementary_Data [file euab021_supplementary_data.pdf]

**Supplementary material**

Tables

Supplementary Table 1: Cost of additional investigations based on the UK NHS tariff

Supplementary Table 2: Further investigations triggered by cardiovascular screening

Supplementary Table 3: Characteristics of individuals identified with cardiovascular disease associated with SCD

Supplementary Table 4: Characteristics of individuals identified with other cardiac disease requiring surveillance

Figures

Supplementary Figure 1: Health questionnaire

**Supplementary Table 1: Cost of additional investigations based on the UK NHS tariff**

| Secondary Investigations | Cost (€) |
|---|---|
| Hospital appointment for consultation following screening *(includes repeat ECG)* | 185 |
| Transthoracic echocardiography | 83 |
| Exercise stress test | 192 |
| Holter | 192 |
| Cardiac magnetic resonance imaging | 237 |
| 24 hour blood pressure monitoring | 192 |
| Signal average ECG | 30 |
| Electrophysiological study (± ablation) | 2,250 |
| Implantable loop recorder | 2,340 |
| Tilt testing | 192 |
| Cardiac computed tomography | 124 |
| Transoesophageal echocardiography | 326 |
| Chest radiograph | 28 |
| Lung function testing | 42 |
| Myocardial perfusion nuclear scan | 419 |
| Provocation testing for Brugada syndrome | 452 |
| Genetic testing: Arrythmogenic right ventricular cardiomyopathy (8 gene panel) | 1,152 |
| Genetic testing: Brugada Syndrome (13 gene panel) | 791 |
| Genetic testing: Catecholaminergic polymorphic ventricular tachycardia (5 gene panel) | 791 |
| Genetic testing: Dilated cardiomyopathy (28 gene panel) | 1,152 |
| Genetic testing: Hypertrophic cardiomyopathy (16 gene panel) | 735 |
| Genetic testing: Long QT syndrome (12 gene panel) | 848 |
| Genetic testing: Marfan syndrome | 961 |

**Supplementary Table 2: Further investigations triggered by cardiovascular screening**

| | HQ<br>N (%)<br>(95% CI) | HQ and ECG<br>N (%)<br>(95% CI) | ECG<br>N (%)<br>(95% CI) | TOTAL<br>N (%)<br>(95% CI) |
|---|---|---|---|---|
| Hospital appointment for consultation | 675 (2.5%)<br>(2.3%-2.7%) | 114 (0.4%)<br>(0.4%-0.5%) | 2,175 (8.1%)<br>(7.8%-8.4%) | 2,964 (11.0%)<br>(10.5%-11.6%) |
| Transthoracic echocardiography | 623 (2.3%)<br>(2.2%-2.5%) | 101 (0.4%)<br>(0.3%-0.5%) | 2,136 (7.9%)<br>(7.6%- 8.3%) | 2,860 (10.6%)<br>(10.1%-11.2%) |
| Exercise stress test | 132[‡] (0.5%)<br>(0.4%-0.6%)<br>- | 37[‡] (0.1%)<br>(0.1%-0.2%)<br>2[†] (0.01%)<br>(0.0%-0.03%) | 314[‡] (1.2%)<br>(1.1%-1.3%)<br>44[†] (0.2%)<br>(0.1%-0.2%) | 483 (1.8%)<br>(1.6%-2.1%)<br>46[†] (0.2%)<br>(0.1%-0.2%) |
| Holter | 155[‡] (0.6%)<br>(0.5%-0.7%)<br>1[†] (0.01%)<br>(0.0%-0.02%) | 41[‡] (0.1%)<br>(0.1%-0.2%)<br>2[†] (0.01%)<br>(0.0%-0.03%) | 291[‡] (1.1%)<br>(1.0%-1.2%)<br>33[†] (0.1%)<br>(0.08%-0.2%) | 487 (1.8%)<br>(1.6%-2.1%)<br>36 (0.1%)<br>(0.09%-0.2%) |
| Cardiac magnetic resonance imaging | 25 (0.09%) | 25 (0.09%) | 183 (0.7%) | 233 (0.9%) |

| | | | | |
|---|---|---|---|---|
| | (0.06%-0.1%) | (0.06%-0.1%) | (0.6%-0.8%) | (0.7%-1.1%) |
| 24 hour blood pressure monitoring | 2 (0.01%)<br>(0.0%-0.03%) | 1 (0.004%)<br>(0.0%-0.02%) | - | 3 (0.01%)<br>(0.0%-0.03%) |
| Signal average ECG | 6 (0.02%)<br>(0.01%-0.05%) | 3 (0.004%)<br>(0.0-0.03%) | 31 (0.1%)<br>(0.09%-0.2%) | 40 (0.2%)<br>(0.1%-0.3%) |
| Electrophysiological study (± ablation) | 3 (0.01%)<br>(0.0-0.03%)<br>- | -<br>-<br> | -<br>28* (0.1%)<br>(0.07%-0.2%) | 3 (0.01%)<br>(0.0-0.03%)<br>28* (0.1%)<br>(0.07%-0.2%) |
| Implantable loop recorder | 2 (0.01%)<br>(0.0%-0.03%) | 3 (0.01%)<br>(0.0-0.03%) | - | 5 (0.02%)<br>(0.01%-0.05%) |
| Tilt testing | 10 (0.04%)<br>(0.02%-0.07%) | 1 (0.004%)<br>(0.0%-0.01%) | - | 11 (0.04%)<br>(0.02%-0.08%) |
| Cardiac computed tomography | 4 (0.01%)<br>(0.0-0.03%) | 1 (0.004%)<br>(0.0%-0.01%) | 2 (0.01%)<br>(0.0%-0.03%) | 7 (0.03%)<br>(0.0%-0.07%) |
| Transoesophageal echocardiography | - | - | 6 (0.02%) | 6 (0.02%) |

| | | | | |
|---|---|---|---|---|
| | | | (0.01%-0.05%) | (0.01%-0.05%) |
| Chest radiograph | 3 (0.01%)<br>(0.0-0.03%) | - | - | 3 (0.01%)<br>(0.0-0.03%) |
| Lung function testing | 1 (0.004%)<br>(0.0%-0.02%) | - | - | 1 (0.004%)<br>(0.0%-0.02%) |
| Myocardial perfusion nuclear scan | - | 1 (0.004%)<br>(0.0%-0.01%) | - | 1 (0.01%)<br>(0.0%-0.02%) |
| Provocation testing for Brugada syndrome | 32 (0.1%)<br>(0.09%-0.2%) | 5 (0.02%)<br>(0.01%-0.05%) | - | 37 (0.1%)<br>(0.1%-0.2%) |
| Genetic testing: | | | | |
| Arrythmogenic right ventricular cardiomyopathy (8 gene panel) | - | - | 3 (0.01%)<br>(0.0-0.03%) | 3 (0.01%)<br>(0.0-0.03%) |
| Brugada Syndrome (13 gene panel) | 6 (0.02%)<br>(0.01%-0.05%) | - | - | 6 (0.02%)<br>(0.01%-0.05%) |
| Catecholaminergic polymorphic ventricular tachycardia (5 gene panel) | 2 (0.01%)<br>(0.0%-0.03%) | - | - | 2 (0.01%)<br>(0.0%-0.03%) |

| | | | | |
|---|---|---|---|---|
| Dilated cardiomyopathy (28 gene panel) | - | - | 3 (0.01%) (0.0-0.03%) | 3 (0.01%) (0.0-0.03%) |
| Hypertrophic cardiomyopathy (16 gene panel) | - | - | 11 (0.04%) (0.02%-0.07%) | 11 (0.04%) (0.02%-0.07%) |
| Long QT syndrome (12 gene panel) | 1 (0.01%) (0.0%-0.02%) | 2 (0.01%) (0.0%-0.03%) | 7 (0.03%) (0.02%-0.06%) | 10 (0.04%) (0.02%-0.08%) |
| Marfan syndrome | 3 (0.01%) (0.0-0.03%) | - | - | 3 (0.01%) (0.0-0.03%) |

Key: * risk stratification and treatment for Wolff-Parkinson-White syndrome ECG pattern, †non-invasive risk stratification indication, ‡ diagnostic indication

## Supplementary Table 3: Characteristics of individuals identified with cardiovascular disease associated with SCD

| Disease | Demographics (Age/Gender) | Health Questionnaire abnormality | ECG abnormality | Diagnostics | Risk stratification | Other tests | Treatment beyond lifestyle advice |
|---|---|---|---|---|---|---|---|
| BrS | 17/F | FH of SCD < 50 years | none | ECHO, Holter, AJM | - | - | - |
| BrS | 32/F | FH of SCD <50 years | none | ECHO, EST, Holter, AJM | - | Gene testing | - |
| BrS | 21/M | FH of SCD < 50 years | none | ECHO, EST, Holter, AJM | - | Gene testing | - |
| CHB | 17/M | FH of PPM at 34 years | none | ECHO, EST, Holter, MRI | - | - | PPM implantation |
| BrS | 24/M | FH of SCD < 50 years | none | ECHO, EST, Holter, AJM | - | Gene testing | - |
| BrS | 26/F | FH of BrS | none | ECHO, EST, Holter, AJM | - | Gene testing | - |
| CPVT | 16/M | Syncope | none | ECHO, EST, Holter | - | Gene testing | Pharmacological treatment |
| BrS | 19/F | FH of SCD < 50 years | none | Holter, AJM | - | - | - |
| BrS | 24/M | FH of SCD < 50 years | none | ECHO, EST, Holter, AJM | - | Gene testing | - |
| LQTS | 22/M | FH of SCD < 50 years | none | EST, Holter | - | Gene testing | Pharmacological treatment |
| CPVT | 18/M | Syncope | none | ECHO, EST, Holter | - | Gene testing | Pharmacological treatment |
| BrS | 29/F | FH of SCD < 50 years | none | ECHO, EST, Holter, AJM | - | Gene testing | - |
| Marfan syndrome (3) | 23/M, 25/F, 31/M | FH of Marfan syndrome | none | ECHO, CXR | - | Gene testing | Pharmacological treatment |
| LQTS | 17/M | FH SCD < 50 years | QTc 480msec, TWI V1-2 | ECHO | EST, Holter | Gene testing | Pharmacological treatment |
| LQTS | 19/F | Syncope | QTc 495msec, TWI V1-2 | ECHO | EST, Holter | Gene testing | Pharmacological treatment |
| ARVC | 18/M | None | Ventricular Ectopy | ECHO, CMR | Holter | Gene testing | Pharmacological treatment + ICD |
| ARVC | 22/M | none | TWI V1-3 | ECHO, SAECG, CMR | Holter | Gene testing | Pharmacological treatment |
| ARVC | 19/F | none | TWI V1-2; Epsilon wave | ECHO,SAECG,CMR | EST, Holter | Gene testing | Pharmacological treatment |
| DCM | 20/F | none | TWI 2,3,avF | ECHO, CMR | EST, Holter | Gene testing | Pharmacological treatment |

| | | | | | | | |
|---|---|---|---|---|---|---|---|
| DCM | 18/F | none | Ventricular Ectopy | ECHO, CMR | EST, Holter | Gene testing | Pharmacological treatment |
| DCM | 22/M | none | LAE; LBBB | ECHO | EST, Holter | Gene testing | Pharmacological treatment |
| HCM | 16/M | none | TWI V1-6 | ECHO, CMR | EST, Holter | - | - |
| HCM | 22/M | none | ST depression 2,3,avF | ECHO | Holter | Gene testing | - |
| HCM | 17/M | none | ST depression 1,V4-6 | ECHO | Holter | Gene testing | Pharmacological treatment |
| HCM | 28/M | none | TWI V1-4 | ECHO, CMR | EST, Holter | Gene testing | - |
| HCM | 21/M | none | TWI 2,3,avF | ECHO, CMR | EST, Holter | - | - |
| HCM | 29/F | none | TWI 2,3,avF, V4-6 | ECHO, CMR | EST, Holter | Gene testing | - |
| HCM | 30/M | none | TWI 2,3,avF | ECHO, CMR | EST, Holter | Gene testing | - |
| HCM | 34/M | none | TWI 1,V5-6; LAD | ECHO | EST, Holter | Gene testing | - |
| HCM | 35/M | none | TWI 2,3,avF,V4-6 | ECHO, CMR | EST, Holter | Gene testing | - |
| HCM | 19/F | none | TWI V1-2, 3,avF | ECHO | Holter | Gene testing | - |
| HCM | 19/M | none | TWI V4-6 | ECHO, CMR | EST, Holter | Gene testing | - |
| HCM | 24/M | none | TWI 1,2,3,avF,V4-6 | ECHO, CMR | EST, Holter | Gene testing | - |
| HCM | 22/M | none | TWI 1,2,3,avF,V4-6 | ECHO, CMR | Holter | Gene testing | - |
| HCM | 16/M | none | TWI 2,3,avF,V4-6 | ECHO | Holter | - | - |
| LQTS | 22/F | none | QTc 511 msec | - | Holter | Gene testing | Pharmacological treatment |
| LQTS | 21/F | none | QTc 491 msec | - | Holter | Gene testing | Pharmacological treatment |
| LQTS | 24/M | none | QTc 527 msec | - | Holter | Gene testing | Pharmacological treatment |
| LQTS | 18/F | none | QTc 480msec | EST | Holter | Gene testing | Pharmacological treatment |
| LQTS | 19/M | none | QTc 530 msec | - | Holter | Gene testing | Pharmacological treatment |
| LQTS | 27/F | none | QTc 490 msec | EST | Holter | Gene testing | Pharmacological treatment |
| LQTS | 21/M | none | QTc 486 msec | EST | Holter | Gene testing | Pharmacological treatment |
| NCCM | 30/F | none | TWI V1-5 | ECHO, CMR | Holter | - | Pharmacological treatment + ICD |

| | | | | | | | |
|---|---|---|---|---|---|---|---|
| NCCM | 28/F | none | ST depression 3,avF,V5-6 | ECHO, CMR | Holter | - | Pharmacological treatment |
| WPW<br>(42) | Mean age: 22<br>31/42 M (74%) | none | Short PR, Delta wave | ECHO (23) | EST (20)<br>Holter (15)<br>EPS (28) | - | Conservative management<br>(16)<br>Ablation treatment (26) |

Key:

AJM: Ajmaline provocation testing; ARVC: Arrhythmogenic right ventricular cardiomyopathy; BrS: Brugada syndrome; CHB: Complete heart block; CPVT: Catecholaminergic polymorphic ventricular tachycardia; CMR; Cardiac magnetic resonance imaging; DCM: Dilated cardiomyopathy; ECHO: Echocardiography; EPS: Electrophysiology study; EST: Exercise stress test; F: Female; FH: Family history; HCM: Hypertrophic cardiomyopathy; ICD: Implantable cardioverter defibrillator; LAD: Left axis deviation; LAE: Left atrial enlargement; LBBB: Left bundle branch block; LQTS: Long QT syndrome; M: Male; NCCM: Non-compaction left ventricular cardiomyopathy; PR: PR interval; PPM: Permanent pacemaker; SAECG: Signal average electrocardiography; SCD: Sudden cardiac death; TWI: T-wave inversions; WPW: Wolff-Parkinson-White (WPW) ECG pattern.

**Supplementary Table 4: Characteristics of individuals identified with other cardiac disease requiring surveillance**

| Disease | N | Method of identification | Diagnostics | Comments |
|---|---|---|---|---|
| Atrial fibrillation | 6 | HQ (2)<br>ECG (4) | ECHO (4)<br>Holter (1) | Cardioversion was performed in 3 individuals<br>Ablation was performed in 1 individual |
| Atrial septal defect | 13 | HQ (2)<br>ECG (11) | ECHO (11)<br>TOE (4) | All individuals managed conservatively with no evidence of pulmonary hypertension. |
| Atrial tachycardia | 2 | HQ (0)<br>ECG (2) | ECHO (2)<br>EPS (1) | One individual treated with ablation |
| AV nodal re-entry tachycardia | 1 | HQ (1)<br>ECG (0) | EST (1)<br>EPS (1)<br>Holter (1) | Individual treated with ablation |
| Bicuspid aortic valve | 26 | HQ (10)<br>HQ/ECG (2)<br>ECG (14) | ECHO (22) | None with >mild aortic valve functional disease<br>None with concomitant aortopathy |
| Cor-triatriatum | 2 | HQ (0) | ECHO (2) | Conservative management |

| | | | | |
|---|---|---|---|---|
| | | ECG (2) | CMR (1) | |
| Hypertension | 5 | HQ (4)<br>HQ/ECG (1)<br>ECG (0) | ECHO (5)<br>CMR (1) | All individuals commenced on antihypertensive agents |
| Mitral valve prolapse | 3 | HQ (1)<br>ECG (2) | ECHO (2) | All individuals conservatively managed |

Key:
CMR; Cardiac magnetic resonance imaging; ECHO: Echocardiography; EST: Exercise stress test; HQ: Health questionnaire; TOE: Trans-oesophageal echocardiography.

**Supplementary Figure 1: Health questionnaire**

-CRY Tel: 01737 363222 web: www.c-r-y.org.uk

CRY Mobile Screening

Health Questionnaire

| **For Office Use Only** | | | |
|---|---|---|---|
| Payment Received: | Questionnaire Checked: | Info box ticked: | Consent Signed: |
| Seen By Doctor: | Follow-up Required: Yes No | | |
| Additional Notes: Result: | | | |

| Patient ID no (for office use only): |
|---|

| **Full Name:** | **Date of Screening:** |
|---|---|

| Parents names if under 16: |
|---|

**Personal Details**

| Home *(correspondence)* address: | | | | Doctors name and Address: |
|---|---|---|---|---|
| **POSTCODE:** | | | | **POSTCODE:** |
| Phone Number: | | | | Phone Number: |
| E-mail: | | | | |
| Date of Birth: | Age: | Gender: | | Main Sport(s): |
| Have you had an ECG test before? | | | | If so, when and where? |
| Are you taking any medication: | | | | If so, please describe? |

**Ethnicity** *(please tick the appropriate box)*

| **White** | **Mixed** | **Black** | **Asian** | **Other** |
|---|---|---|---|---|
| British □ | White and Black Caribbean □ | Caribbean □ | Indian □ | Chinese □ |
| Irish □ | White and Black African □ | East African □ | Pakistani □ | Filipino □ |
| Turkish /Cypriot □ | White and Asian □ | West African □ | Bangladeshi □ | Vietnamese □ |
| Greek /Cypriot □ | | North African □ | | Other □ |
| If other, please state your ethnic origin: | | | | |

| **Height ……….cm** | **Weight ………..Kg** |
|---|---|

**1. Have you ever fainted?**

| | | | |
|---|---|---|---|
| During Exercise | Yes / No | How recently did this occur? | If yes, please describe the circumstances |
| Following Exercise | Yes / No | How recently did this occur? | |
| Unrelated to exercise | Yes / No | How recently did this occur? | |

**2. Do you experience dizzy turns?**

| | | | |
|---|---|---|---|
| During Exercise | Yes / No | How recently did this occur? | If yes, please describe the circumstances |
| Following Exercise | Yes / No | How recently did this occur? | |
| Unrelated to exercise | Yes / No | How recently did this occur? | |

**Do you experience palpitations?** *(palpitations are a fluttering in your chest that you can notice whilst resting)*

| | |
|---|---|
| Yes / No | If yes, how recently and please describe the circumstances |

**3. Do you experience chest pain, heaviness or tightness?**

| | | |
|---|---|---|
| During Exercise | Yes / No | If yes, please describe the circumstances |
| Following Exercise | Yes / No | |
| Unrelated to exercise | Yes / No | |

**4. Do you feel that you are more breathless or more easily tired than your team mates?**

| | |
|---|---|
| Yes / No | If yes, please describe the circumstances |

**5. Is there a family history of heart disease in anyone under the age of 50?**

| | |
|---|---|
| Yes / No | If yes, how are they related to you, what is the diagnosis? Please state the age of onset |

**6. Has anyone died suddenly in your family under the age of 50?**

| | |
|---|---|
| Yes / No | If yes, how were they related to you? Please describe the circumstances and at what age did the death occur |

**7. Approximately, how many days per week are you physically active (playing sport)? ……………**

**8. On average, how many hours per week are you physically active (playing sport)? …….........**

**9. If you are competitive athlete what sports do you play and at what level?**

| | | |
|---|---|---|
| e.g. International, National, County, | (Main sport)………………………. | Level: ………………………………… |
